# Supplementary figures and images for: A fluorescent plasmonic biochip assay for multiplex screening of diagnostic serum antibody targets in human Lyme disease
Source: PLoS One. 2020 Feb 10;15(2):e0228772. doi: 10.1371/journal.pone.0228772 (PMC7010292; doi:10.1371/journal.pone.0228772)

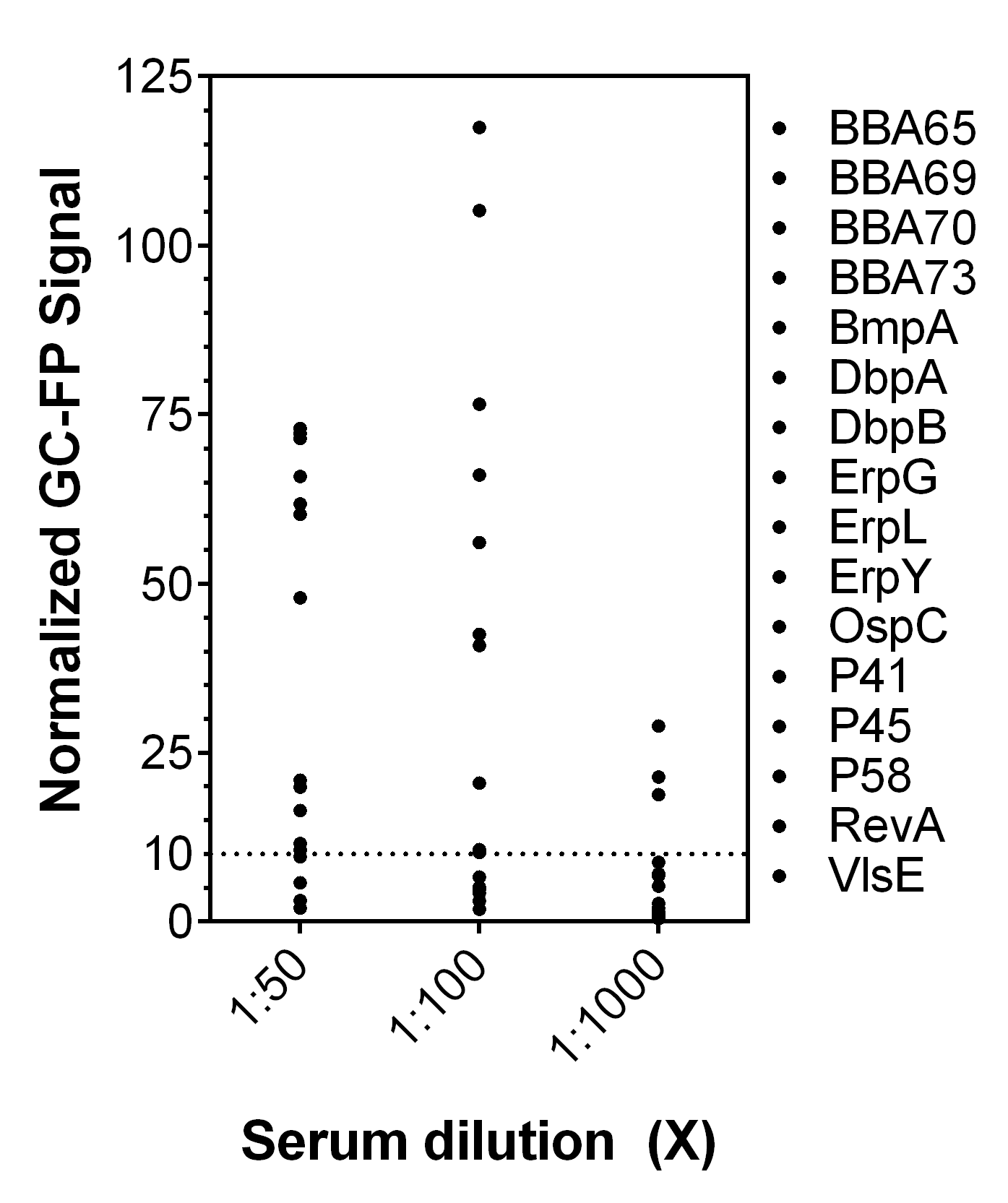

Supplement: S1 Fig — Increasing concentrations of a pooled LD positive control serum (1:1000 X, 1:100 X, and 1:50 X dilutions) were flowed across a biochip spotted with 16 different antigens. The mean normalized GC-FP signal binding of IgG to each antigen is plotted. The highest signals were observed at 1:100 X serum dilution, in which 8 out of 16 targets had signal above 10 (arbitrary units). (TIF) [file pone.0228772.s001.tif]

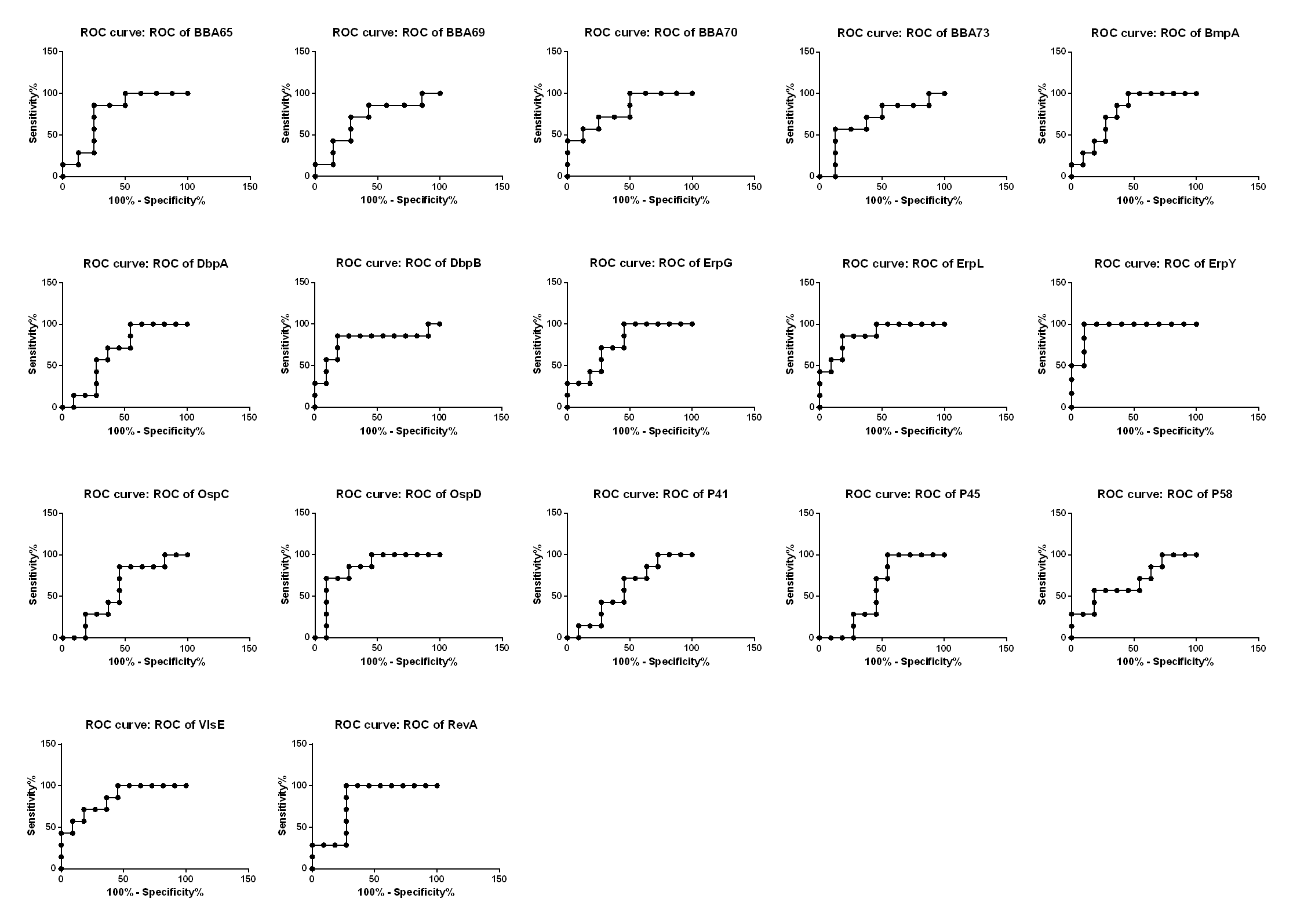

Supplement: S2 Fig — Data from 20 LD-positive and 11 negative control serum samples were included in a training set to generate ROC curves evaluating the independent predictive abilities of 17 potential diagnostic targets: DbpA, P58, RevA, BBA65, BmpA, P41, ErpL, BBA69, VlsE, DbpB, ErpY, BBA70, OspC, P45, ErpG, BBA73, and OspD. (TIF) [file pone.0228772.s002.tif]
