# Supplementary material for: A fluorescent plasmonic biochip assay for multiplex screening of diagnostic serum antibody targets in human Lyme disease
Source: PLoS One. 2020 Feb 10;15(2):e0228772. doi: 10.1371/journal.pone.0228772 (PMC7010292; doi:10.1371/journal.pone.0228772)
Supplement: Attachment — Submitted filename: Response to Reviewers.docx [file pone.0228772.s004.docx]

29 December 2019

To: Sabato D’Auria, Academic Editor, PLoS One

RE: PONE-D-19-27828 “A fluorescent plasmonic biochip assay for multiplex screening of diagnostic serum antibody targets in human Lyme disease”

Dr. D’Auria –

Thank you for sending the reviews of our manuscript. We are pleased with the reviewers’ responses and the decision to accept the manuscript with minor revisions. We have edited the manuscript as per the reviewer comments and provide the following point by point responses:

1. When submitting your revision, we need you to address these additional requirements. Please ensure that your manuscript meets PLOS ONE's style requirements…

*We have checked these additional style requirements and made sure to update the manuscript accordingly.*

2. Thank you for including the following ethics statement on the submission page: 'The use of human blood serum samples for this study has been approved by the SUNY Polytechnic Institute IRB. Human serum samples were obtained from the Lyme Disease Biobank, Centers for Disease Control, and NY State Department of Health. All samples were de-identified and thus analyzed anonymously. Samply collection by the original collection agencies was performed with written consent.'
   Please also include this ethics statement in the Methods section of your manuscript.

*We have added the ethics statement to the methods section of the manuscript:*

The use of human blood serum samples for this study was approved by the SUNY Polytechnic Institute IRB. Human serum samples were obtained from the Lyme Disease Biobank, Centers for Disease Control, and from Dr. Susan Wong (NY State Department of Health). All samples were de-identified and thus analyzed anonymously. Sample collection by the original collection agencies was performed with written consent.

3. PLOS requires an ORCID iD for the corresponding author in Editorial Manager on papers submitted after December 6th, 2016. Please ensure that you have an ORCID iD and that it is validated in Editorial Manager.

*Done.*

4. Thank you for stating the following in the Financial Disclosure  section: "This work was supported by National Science Foundation IOS1755286 (Y.L. and B.T.), National Science Foundation DBI1757170 (B.T.), Department of Defense TB170111 (Y.L. and B.T.), New York State Department of Health Wadsworth Center Start-Up Grant (Y.L. and B.T.), SUNY Polytechnic Faculty Seed Award (N.C.). "We note that one or more of the authors are employed by a commercial company: name of commercial company.

Please provide an amended Funding Statement declaring this commercial affiliation, as well as a statement regarding the Role of Funders in your study. If the funding organization did not play a role in the study design, data collection and analysis, decision to publish, or preparation of the manuscript and only provided financial support in the form of authors' salaries and/or research materials, please review your statements relating to the author contributions, and ensure you have specifically and accurately indicated the role(s) that these authors had in your study. You can update author roles in the Author Contributions section of the online submission form……..

*The statement in the acknowledgements section was modified as follows, to provide a clearer statement on the role of funding agencies and the authors who were employed by a commercial entity, Ciencia, Inc. during the study:*

This work was supported by National Science Foundation IOS1755286 (Y.L. and B.T.), National Science Foundation DBI1757170 (B.T.), Department of Defense TB170111 (Y.L. and B.T.), New York State Department of Health Wadsworth Center Start-Up Grant (Y.L. and B.T.), SUNY Polytechnic Faculty Seed Award (N.C.). Funding agencies did not play a role in the study design, data collection and analysis, decision to publish, or preparation of the manuscript. Ciencia, Inc. provided support in the form of salaries for authors A.P., E.G., and W.P., but did not have any additional role in the study design, data collection and analysis, decision to publish, or preparation of the manuscript. The specific roles of these authors are articulated in the ‘author contributions’ section. The authors have no conflict of interest to declare.

5. Please also provide an updated Competing Interests Statement declaring this commercial affiliation along with any other relevant declarations relating to employment, consultancy, patents, products in development, or marketed products, etc.

*Updated Competing Interests Statement:*

Authors A.P., E.G., and W.P. were employed by Ciencia, Inc. during the course of this study. This does not alter our adherence to PLOS ONE policies on sharing data and materials.

6. Please include captions for your Supporting Information files at the end of your manuscript, and update any in-text citations to match accordingly. Please see our Supporting Information guidelines for more information: <http://journals.plos.org/plosone/s/supporting-information>.

*The supporting information figure captions were included in the original submission as “Supplementary Files”. This section has been re-labelled as “Supporting Information.” The actual files have been uploaded separately according to submission guidelines.*

7. *Some minor changes were made per co-author/collaborator requests. These are highlighted in yellow in the track changes version of the manuscript text and include: (1) Update to Benjamin Taubner’s affiliation in the author list, (2) Alternative labelling of the CDC samples from numbers to letters according to the materials transfer agreement with the CDC, (3) Additional acknowledgements, (4) Figures updated to reflect new Fig. 2 and edited Fig. 5 to clarify combined IgG + IgM signal in the legend.*
8. **Reviewer #1:** The manuscript by Nathaniel C. Cady et al. ……..The aim of this work is interesting and the results are promising. The paper is convincing and well done in almost all its parts. Some restructuration of the text may apply to the methods section. In the present form, the paper demands a revision before it can be published, so this reviewer suggests a “minor revision”.
   ………This reviewer thinks that could be useful add a subsection in the Methods section that describes better (In details) the assay. The authors reported many references, but an image or a scheme could help the reader to understand better the method proposed and developed.

*We thank the reviewer for his/her positive comments and have added a figure to the methods section that shows the overall scheme of the biosensing / biosensor platform and the assay that was used for this work. The manuscript text was also modified to describe and refer to the figure.*

9. **Reviewer #2:** The manuscript by Eunice Chou et al. ……In my opinion the reading of the work is very fluent, the data are well organized. I admit this manuscript, even if I believe that these studies still have points to be strengthened, good results have been achieved.

*We thank the reviewer for his/her positive comments!*

Sincerely,

Nathaniel C. Cady, PhD

Corresponding Author
